# Switchable multifunctional terahertz metasurfaces employing vanadium dioxide

**DOI:** 10.1038/s41598-019-41915-6

**Published:** 2019-04-01

**Authors:** Xike Li, Shiwei Tang, Fei Ding, Shuomin Zhong, Yuanqing Yang, Tao Jiang, Jun Zhou

**Affiliations:** 1 Department of Physics, Faculty of Science, Ningbo University, Ningbo, 315211 China; 2 SDU Nano Optics, University of Southern Denmark, Campusvej 55, Odense, DK-5230 Denmark; 3 Faculty of Electrical Engineering and Computer Science, Ningbo University, Ningbo, 315211 China

## Abstract

In this paper, we design a type of switchable metasurfaces by employing vanadium dioxide (VO_2_), which possess tunable and diversified functionalities in the terahertz (THz) frequencies. The properly designed homogeneous metasurface can be dynamically tuned from a broadband absorber to a reflecting surface due to the insulator-to-metal transition of VO_2_. When VO_2_ is in its insulating state, the metasurface can efficiently absorb the normally incident THz wave in the frequency range of 0.535–1.3 THz with the average absorption of ~97.2%. Once the VO_2_ is heated up and switched to its fully metallic state, the designed metasurface exhibits broadband and efficient reflection (>80%) in the frequency range from 0.5 to 1.3 THz. Capitalizing on such meta-atom design, we further extend the functionalities by introducing phase-gradients when VO_2_ is in its fully metallic state and consequently achieve polarization-insensitive beam-steering and polarization-splitting, while maintaining broadband absorption when VO_2_ is in insulating state.

## Introduction

The capability of manipulating electromagnetic (EM) waves is of critical importance to many EM-based devices and systems in both fundamental and applied sciences. However, the typical EM devices relying on the gradually accumulated phase during wave propagation are suffering from curved or spatially-distributed shapes and bulky configurations, which limit their potential applications in EM integration for the ever-increasing demands on data-storage capacity and information processing speed. Additionally, EM devices that possess multiple diversified functionalities yet with miniaturized configurations are greatly desired. In recent years, metasurfaces, the two-dimensional analogues of metasurfaces that consist of planar arrays of nanostructures, have become an emerging research area due to their unprecedented control over scattered EM fields within the compact and ultrathin geometries, thereby releasing the nano-fabrication and decreasing the loss to some extent^1–4^. By designing artificial meta-atoms and positioning them properly in a periodic or aperiodic manner, arbitrary optical responses with different local amplitudes, phases, and polarizations can be achieved. Therefore, a variety of novel physics phenomena and attractive EM devices have been demonstrated with metasurfaces, such as generalized Snell’s law^5–14^, surface waves coupling^15–18^, focusing lenses^19–22^, holograms^23–25^, absorbers^26,27^, as well as polarization generation and detection^28–34^. Besides the surface-confined configurations, compact footprints and arbitrary wavefront control, metasurfaces are capable of effectively integrating multiple distinct functionalities into one single device^35–44^, superior to the aforementioned metasurfaces with single functionality.

However, in most of these approaches, the metasurfaces possess fixed functionalities once been fabricated, lacking the switchable ability and thus hindering practical applications. As such, it is highly desired to achieve switchable metasurfaces that are capable of integrating two or more diversified functionalities together and actively tuning different functionalities at the same time. To realize switchable metasurfaces, an effective way is to incorporate standard metasurfaces with phase-change materials (PCMs), such as chalcogenide GeSbTe(GST) alloys^45–54^, vanadium dioxide (VO_2_)^55–71^. In spite of the significant achievements in PCMs integrated metasurfaces with dynamic responses, switchable metasurfaces exhibiting totally different functionalities that can be actively switched over a wide frequency range are still largely unexplored.

In this paper, we propose a type of switchable metasurfaces by employing the insulator-to-metal transition in VO_2_, which possess tunable and diversified functionalities in the terahertz (THz) frequencies. The designed homogeneous metasurface can be dynamically tuned from a broadband absorber to a reflecting surface by thermally stimulus. At room temperature, VO_2_ is an insulator and the metasurface can efficiently absorb the incident THz wave at normal incidence in the frequency range of 0.535–1.3 THz with the average absorption as high as ~97.2%. If VO_2_ is heated up and switched to its fully metallic state, the metasurface could reflect the incident wave efficiently and the reflectivity is above 80% in the frequency spectrum ranging from 0.5 to 1.3 THz. Furthermore, we introduce phase-gradients by varying the topmost VO_2_ antennas of the metasurface when VO_2_ is in its fully metallic state and consequently realize beam-steering functionalities. Meanwhile, the metasurface can work as an efficient absorber when VO_2_ is in its insulating state.

## Design of the Switchable Multifunctional THz Metasurfaces

**Figure 1:**
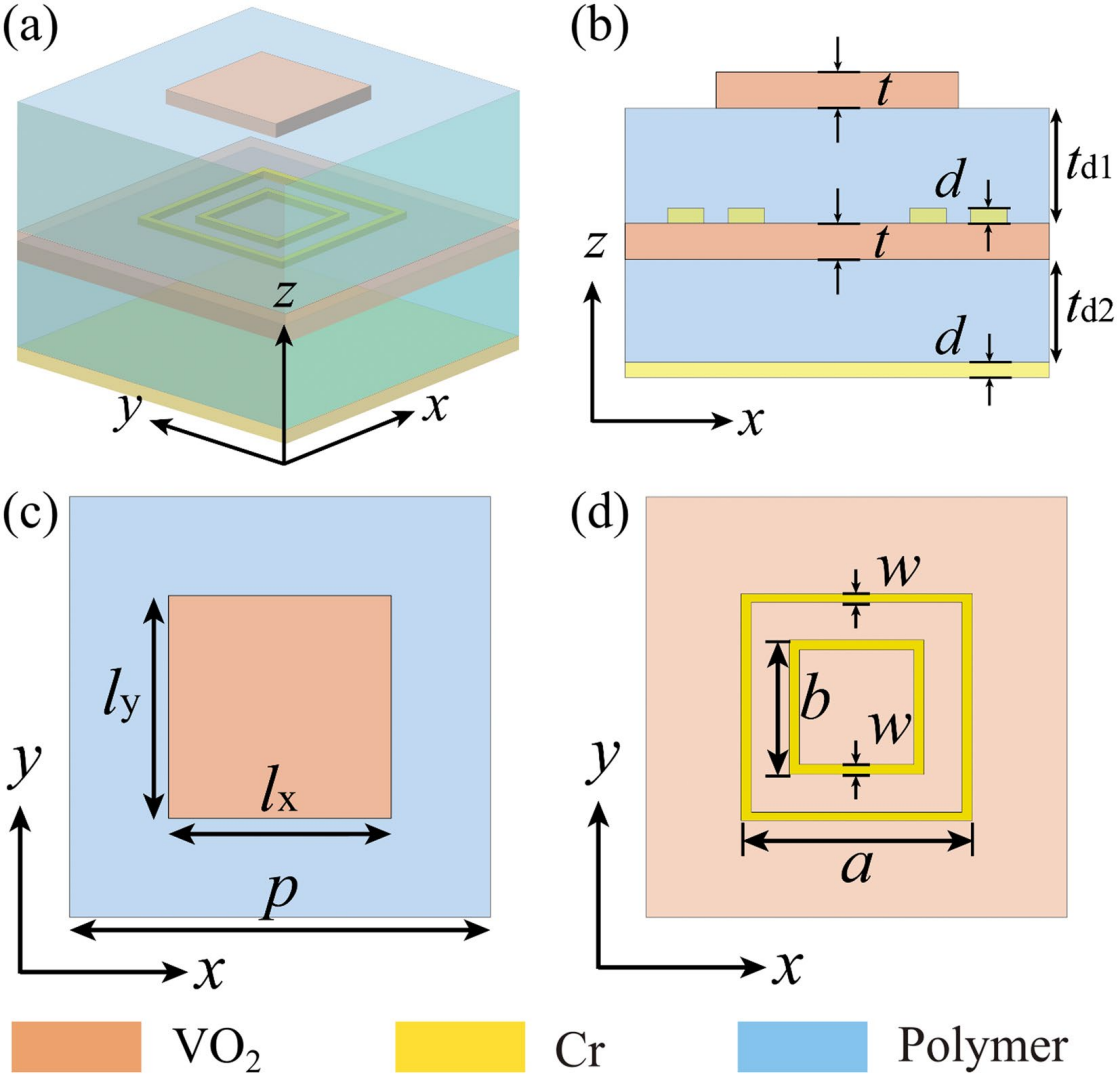
(**a**) 3D schematic of the unit cell of proposed switchable multifunctional THz metasurfaces employing VO_2_. (**b**) Side view of the unit cell composed of six functional layers. (**c,d**) Top view of the unit cell in different cross-sections. The geometrical dimensions are *p* = 90 μm, *l*_x_ = *l*_y_ = 30 μm, *t* = 1 μm, *t*_d1_ = 30 μm, *t*_d2_ = 34 μm, *a* = 55 μm, *b* = 36 μm, *w* = 1 μm, and *d* = 0.3 μm, respectively.

Figure 1 illustrates the basic unit cell of the VO_2_ integrated THz metasurfaces, which is composed of six different functional layers. Specifically, the functional layers from top to bottom are periodic VO_2_ brick-shaped antennas, a polyimide spacer layer, double chrome (Cr) square ring resonators (SRRs), a continuous VO_2_ layer, a second polyimide layer and a bottom Cr substrate (Fig. 1(b)). At room temperature, VO_2_ is an insulator with a low conductivity of *σ* = 200 S/m^56^. In this regard, the unit cell is equivalent to a dielectric-covered metal-insulator-metal (MIM) resonator that consists of Cr SRRs, the second polyimide spacer, and the bottom Cr substrate. Therefore, the incident THz wave can transmit through the topmost VO_2_ brick array and interact with Cr SRRs, resulting in highly-efficient absorption. On the other hand, once the temperature is increased above the phase-change temperature of *T*_c_ = ~340 K, VO_2_ undergoes an insulator-to-metal transition and gains a high conductivity^56^. When VO_2_ is in its fully metallic state, the conductivity *σ* can reach as high as 2 × 10^5^ S/m^59,61^. In this case, the VO_2_ brick-shaped antennas interact strongly with the THz wave and the continuous VO_2_ film can block all the transmission, thereby forming another MIM cavity composed of VO_2_ brick array, the polyimide spacer and the VO_2_ continuous film. By tailoring the dimensions of such MIM cavity in which VO_2_ functions as metal, one can realize reflecting surface and consequently manipulate the reflected wave at will.

To begin with, we analyze a three-dimensional (3D) homogeneous metasurface composed of periodic meta-atoms shown in Fig. 1(a) by using the commercially available software Comsol Multiphysics (ver. 5.3) based on finite element method (FEM) to determine the reflection coefficients. Periodic boundaries are applied in both the *x*- and *y*-directions, and the medium above the unit cell is chosen to be air and truncated using a perfectly matched layer (PML) to minimize reflection. An *x*- or *y*-polarized plane wave is normally incident on the top surface as the excitation source along the -*z*-direction. In our simulations, the relative permittivity of VO_2_ is described by Drude model $\varepsilon (\omega )={\varepsilon }_{\infty }-\frac{{\omega }_{p}^{2}\frac{\sigma }{{\sigma }_{0}}}{{\omega }^{2}+i\cdot \omega \cdot {\omega }_{d}}$ with epsilon infinity *ε*_∞_ = 12, the plasma frequency *ω*_p_ = 1.4 × 10^15^ s^−1^, the damping frequency *ω*_d_ = 5.75 × 10^13^ s^−1^, and *σ*_0_ = 3 × 10^5^ S/m^59,61^. The conductivity *σ* of VO_2_ is 200 S/m and 2 × 10^5^ S/m in insulating and fully metallic states corresponding to the temperature of ~298 K (room temperature) and ~358 K, respectively^59,61^. The thickness of VO_2_ structures is *t* = 1 μm. The thickness of Cr SRRs is *d* = 0.3 μm and the conductivity of Cr is set to be 2.2 × 10^5^ S/m^72^. The polyimide spacer layer is considered to be a lossy dielectric with a constant relative permittivity of *ε* = 2.4 + 0.005i. The other geometrical dimensions are *p* = 90 μm, *l*_x_ = *l*_y_ = 30 μm, *t*_d1_ = 30 μm, *t*_d2_ = 34 μm, *a* = 55 μm, *b* = 36 μm, and *w* = 1 μm, respectively.

**Figure 2:**
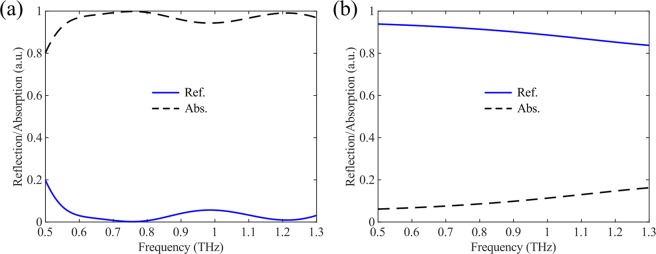
(**a**) Simulated absorption and reflection of the homogeneous metasurface at normal incidence when VO_2_ is in its insulating state with *σ* = 200 S/m. (**b**) Simulated absorption and reflection of the homogeneous metasurface at normal incidence when VO_2_ is in its fully metallic state with *σ* = 2 × 10^5^ S/m.

Figure 2 shows the simulated absorption and reflection spectra when VO_2_ is in different states. From Fig. 2(a), it is clearly observed that this VO_2_ incorporated metasurface can efficiently absorb the normally incident THz wave over an ultra-broadband spectrum when VO_2_ is in its insulating state at room temperature. In particular, the calculated absorption is exceeding 90% in the frequency range of 0.535–1.3 THz with the average absorption reaching ~97.2%. Such broadband and efficient absorption is ascribed to the bottom MIM cavity formed by the Cr SRRs, the continuous VO_2_ film, the second polyimide spacer and the bottom continuous Cr film, which can interact effectively with the incident wave. It is worth noticing that such MIM cavity can also be regarded as an asymmetric Fabry–Perot resonator^73^. When the THz wave impinges on the structure, the interface between Cr SRRs and the continuous VO_2_ film induces significant reflection/transmission phase. Thus, the conventional Fabry–Perot resonance condition will be modified as the propagation phase within the dielectric layer is no longer dominating.

**Figure 3:**
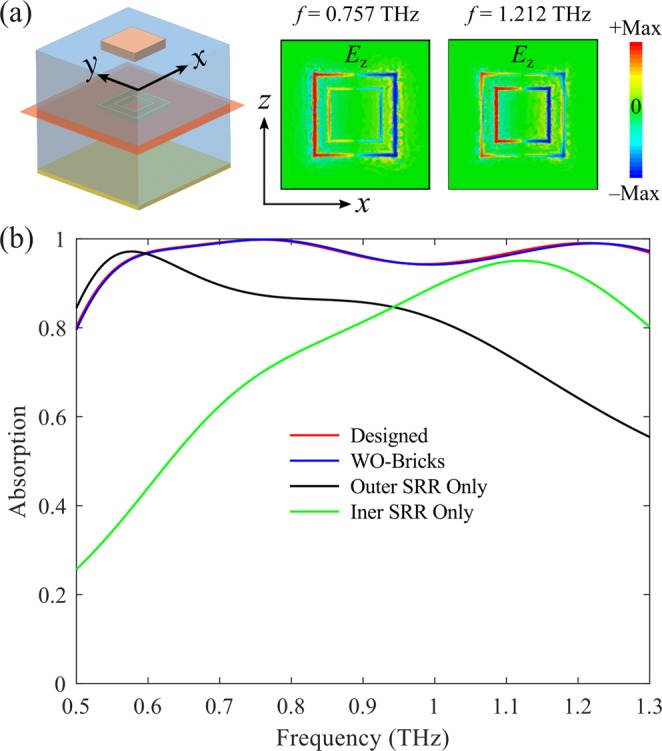
Mechanism of the broadband absorption when VO_2_ is in its insulating state with *σ* = 200 S/m. (**a**) Normalized *E*_z_ distributions of the two absorption peaks at *f* = 0.757 and 1.212 THz in the *x-y* plane. (**b**) Simulated absorption spectra of different configurations.

To verify the underlying mechanism for wideband absorption, we plot the field distributions of the two absorption peaks at *f* = 0.757 and 1.212 THz, respectively (Fig. 3(a)). When the THz wave impinges on the metasurface, it can firstly transmit through the topmost VO_2_ brick array which is transparent when VO_2_ is an insulator at room temperature (Fig. 3(b)). After that, the incident wave interacts strongly with the MIM cavity, especially the Cr SRRs, resulting in electric dipole resonances. Similar to the previous work^61^, the induced dipole resonances at certain frequencies are related to the SRRs’ arms that are parallel to the electric field of incident wave. Therefore, the two absorption peaks correspond to the resonances of the outer and inner SRRs, respectively. For instance, at *f* = 0.757 THz, the incident wave is mainly confined around the outer SRR with bigger size while the electromagnetic field near the inner SRR is weak. Once the inner or outer SRR is removed, the absorption becomes narrower with only one main peak. However, due to the coupling between inner and out SRRs, the resonance peak supported by single-sized SRR is not totally matched with the corresponding peak of double-sized SRRs, as shown in Fig. 3(b). Additionally, since Cr has more loss than other noble metals, such as gold and silver, these resonances have rather low quality-factor and they can merge together easily, thereby broadening the absorption bandwidth^72^.

Once the temperature increases gradually, the conductivity of VO_2_ increases and the absorption decreases. If the temperature is kept above 358 K, VO_2_ is switched to its fully metallic state and the designed unit cell transits to another MIM cavity consisting of topmost VO_2_ brick array, the polyimide spacer, and the VO_2_ continuous film, thus allowing for high reflection instead of absorption (Fig. 2(b)). As shown in Fig. 2(b), broadband and efficient reflection (>80%) is sustained in the frequency range from 0.5 to 1.3 THz when VO_2_ becomes fully metallic, distinct from the case of broadband absorption when VO_2_ is in the insulating state. Therefore, the designed VO_2_ integrated THz metasurface possesses switchable multiple functionalities, which can be switched from a broadband absorber to an efficient reflector by exploiting the temperature-controlled phase transition in VO_2_.

## Switchable Multifunctional THz Metasurfaces with Phase-gradients

**Figure 4:**
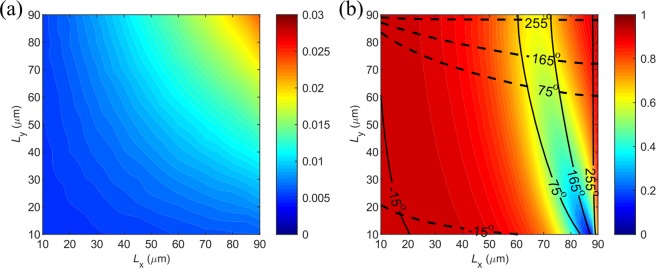
Calculated reflection as a function of the VO_2_ brick dimensions at the design frequency of *f* = 0.8 THz under *x*-polarization when VO_2_ is (**a**) in the insulating state with *σ* = 200 S/m and (**b**) in its fully metallic state with *σ* = 2 × 10^5^ S/m. The reflectivity map is for *x*-polarization, while the lines are contours of the reflection phases for both *x*-polarization (solid lines) and *y*-polarization (dashed lines). Note that the reflectivity for *y*-polarization can be obtained by mirroring the map for *x*-polarization along the line *l*_x_ = *l*_y_.

In addition to the switchable functionalities between broadband absorption and highly-efficient reflection with a homogeneous metasurface utilizing single meta-atom, the proposed metasurface can be further engineered with phase-gradients, thereby allowing for arbitrary beam-steering of the reflected THz wave when VO_2_ is in its fully metallic state while maintaining wideband absorption with insulating VO_2_. To achieve phase-gradients, we have calculated the reflection coefficients of each meta-atom at the design frequency of *f* = 0.8 THz by varying the lateral dimensions of the meta-atom (*l*_x_ and *l*_y_) while the other parameters are kept fixed. As shown in Fig. 4(a), when VO_2_ is in its insulating state, the reflectivity is sufficiently low (less than 2%) regardless of the varied topmost VO_2_ bricks with different dimensions, which is consistent with the previous discussion that the absorption is mainly determined by the SRRs-polymer-Cr cavity. In contrast to the ultra-low and nearly-constant reflectivity when VO_2_ is in the insulating state at room temperature, the reflectivity and phase response of each unit cell can be engineered by changing the dimensions of VO_2_ bricks at *f* = 0.8 THz when VO_2_ is in its fully metallic state (Fig. 4(b)). For example, the reflectivity can be greatly modulated by *l*_x_ while *l*_y_ has little effect on *x*-polarized THz wave at *f* = 0.8 THz, which is ascribed to the strong absorption induced by the gap-surface plasmon (GSP) resonance within the VO_2_-polyimide-VO_2_ cavity. Meanwhile, the two degrees of freedom (dimensions *l*_x_ and *l*_y_) allow us to vary the phases of reflected polarizations approximately within the whole phase space of ~2π^12,44^. Close to the GSP resonance, the reflection phase varies rapidly over the range of π. Away from the resonance, the reflection phase is affected by the retardation of the incident wave when being reflected by the bottom reflected or by the process of coupling into GSPs. Here it should be mentioned that for larger *l*_x_ the reflection phase of *x*-polarized wave does depend on the value of *l*_y_, which is ascribed to the coupling between neighboring elements. Therefore, one should consider this dependence when designing phase-gradients.

**Figure 5:**
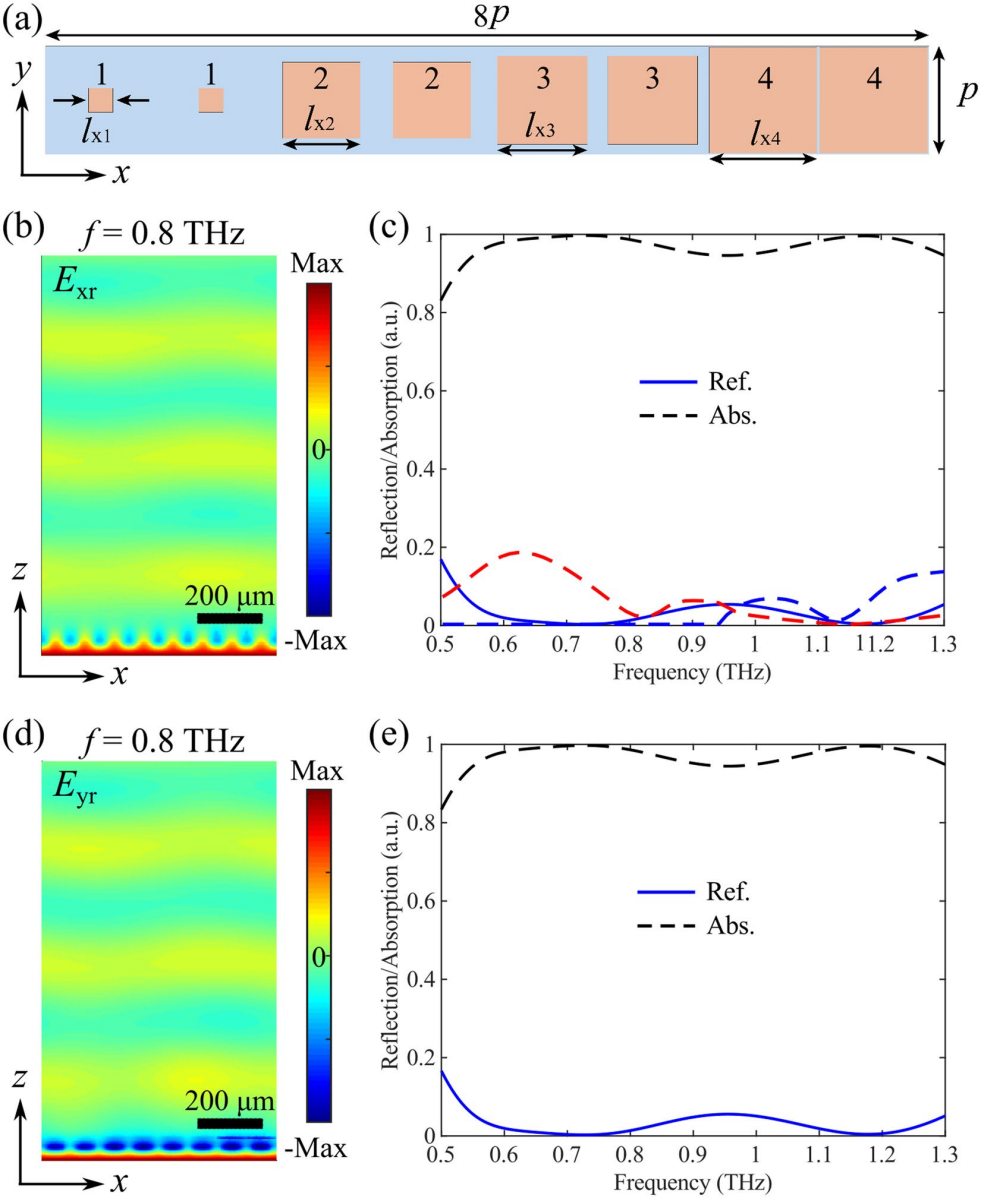
(**a**) Supercell of the polarization-insensitive switchable multifunctional THz metasurface that functions as a broadband absorber. The dimensions are *l*_x1_ = 20 μm, *l*_x2_ = 64 μm, *l*_x3_ = 73.5 μm and *l*_x4_ = 88 μm. (**b,c**) Theoretical performance of the metasurface for *x*-polarization, displaying b) the *x*-component of the reflected electric field (*E*_rx_) at the design frequency of *f* = 0.8 THz, and (**c**) reflection and absorption spectra as a function of the frequency. (**d,e**) Theoretical performance of the metasurface for *y*-polarization, displaying d) the *y*-component of the reflected electric field (*E*_ry_) at the design frequency of *f* = 0.8 THz, and (**e**) reflection and absorption spectra as a function of the frequency. VO_2_ is in its insulating state with *σ* = 200 S/m.

In order to extend the functionalities, we first integrate several isotropic VO_2_ bricks in a supercell to create identical linear phase-gradients for both *x*- and *y*-polarizations along the *x*-direction when VO_2_ is in its metallic state. In this case, we can achieve polarization-insensitive anomalous reflection while maintaining broadband efficient absorption when VO_2_ is in its insulting state. Figure 5(a) illustrates the schematic of the supercell consisting of eight elements with a center-to-center distance of *p* = 90 μm, where four different square-shaped meta-atoms are selected from the intersection points between the solid and dashed lines in Fig. 4(b). In this regard, every meta-atom is arranged in pairs and the constructed supercell has a lateral period of 720 μm along *x*-direction, which is large enough to avoid generating driven surface waves^15^. When VO_2_ is an insulator at room temperature, the selected four meta-atoms have rather low reflectivity and the reflection phase plays little effect on the reflected field. Therefore, the resulting supercell can efficiently absorb the incident THz wave at the design frequency of *f* = 0.8 THz for *x*-polarization with the absorption reaching as high as 98.7% (Fig. 5(b)). Additionally, the high-performance absorption is sustained over a wide frequency band. For instance, the calculated absorption is exceeding 83% from 0.5 THz to 1.3 THz, and the average absorption is ~97.1%, as shown in Fig. 5(c). Furthermore, the supercell exhibits polarization-insensitive absorption due to the selected isotropic meta-atoms that have identical responses for two linear polarizations (Fig. 5(d,e)).

**Figure 6:**
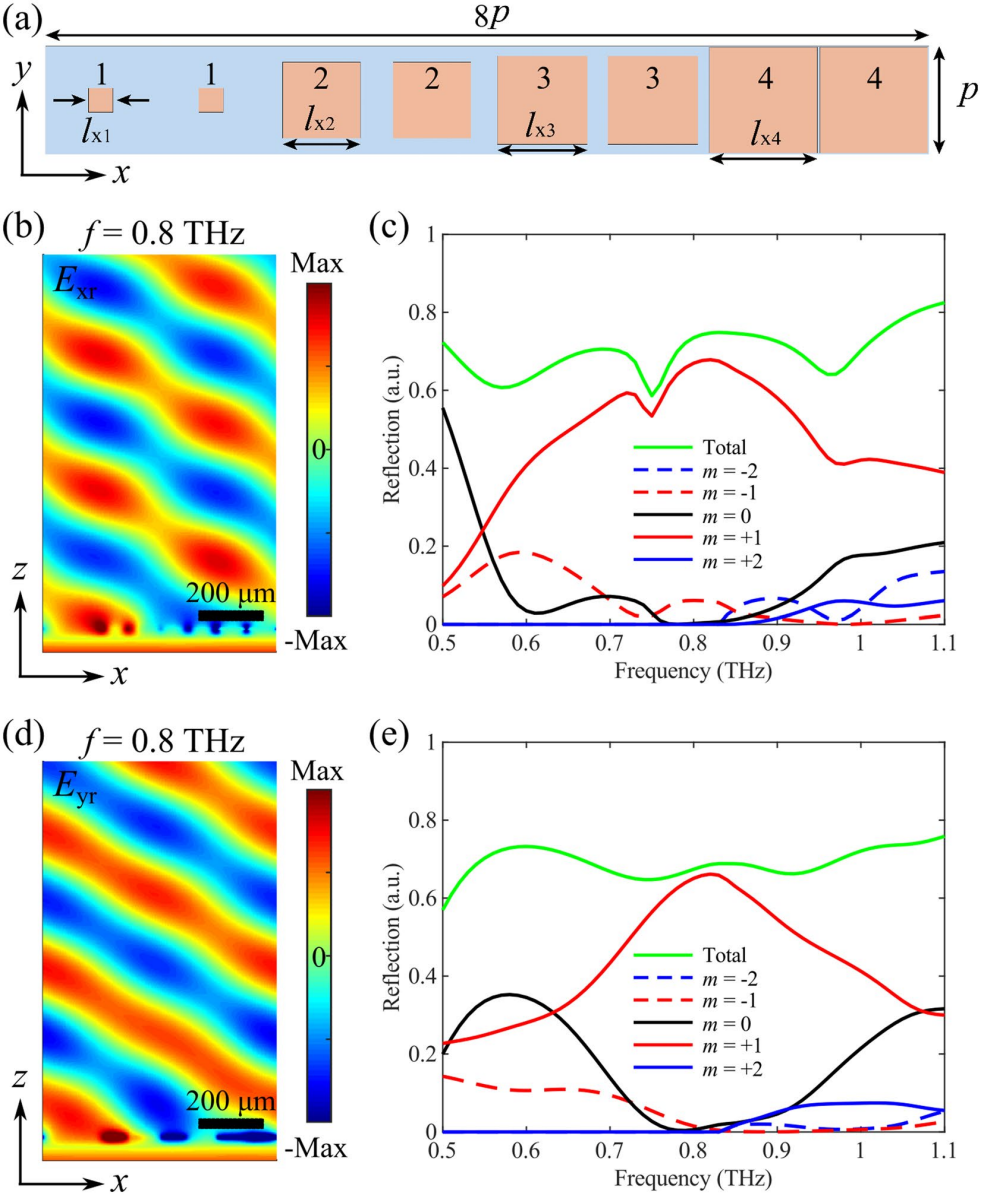
(**a**) Supercell of the polarization-insensitive switchable multifunctional THz metasurface that functions as a broadband beam-steerer. The dimensions are *l*_x1_ = 20 μm, *l*_x2_ = 64 μm, *l*_x3_ = 73.5 μm, and *l*_x4_ = 88 μm. The reflection phases of the meta-atoms 1–4 are −14.5°, 75.7°, 165.3°, and 254.9°, respectively, for both *x*- and *y*-polarizations. (**b,c**) Theoretical performance of the metasurface for *x*-polarization, displaying (**b**) the *x*-component of the reflected electric field (*E*_rx_) at the design frequency of *f* = 0.8 THz, and (**c**) amount of incident THz wave reflected into the different diffraction orders as a function of the frequency. (**d,e**) Theoretical performance of the metasurface for *y*-polarization, displaying (**d**) the *y*-component of the reflected electric field (*E*_ry_) at the design frequency of *f* = 0.8 THz, and (**e**) amount of incident THz wave reflected into the different diffraction orders as a function of the frequency. VO_2_ is in its fully metallic state with *σ* = 2 × 10^5^ S/m.

Distinct from the insulating state, the selected four meta-atoms have considerably high reflectivity and the reflection phase can be utilized to mold the reflected fields when VO_2_ is in its fully metallic state, according to the generalized Snell’s law^5^. In particular, the supercell with eight elements provides a polarization-insensitive 2π phase span with a phase step of π/2 for two incident linear polarizations along the *x*-direction, thus enabling polarization-independent beam-steering for the reflected THz wave with the anomalous reflection peak appearing at an angle of ~31.4°. In order to verify the polarization-insensitive beam-steering, 3D full-wave simulations were conducted by modeling the periodic supercell shown in Fig. 6(a). Figure 6(b) displays the reflected electric filed *E*_xr_ at *f* = 0.8 THz for *x*-polarization when VO_2_ is in its fully metallic state, indicating the well-defined wavefront of a plane wave. In addition, the +1 diffraction order is dominating while other diffraction orders are greatly suppressed (Fig. 6(c)). Specifically, over 91.1% of the reflected light is routed to the +1 diffraction order and the absolute reflectivity reaches 67.0%. Given that around 26.5% of the incident THz light is absorbed when VO_2_ is in fully metallic state, the achieved diffraction efficiency in the desired direction is moderately high^74–76^. Here it is worth noting that though the 0 diffraction order is almost totally suppressed, the unwanted −1 diffraction order shows up at the design frequency, which may be ascribed to the variations in reflectivity produced by different elements comprising the supercell and the near-field coupling between different meta-atoms^77^.

As expected, the *y*-polarized THz wave is also reflected to the +1-diffraction order (Fig. 6(d,e)). Compared to the performance of beam-steering for *x*-polarization, the reflected wave of *y*-polarization shows fewer distortions, assembling a better wavefront closer to a plane wave, as shown in Fig. 6(d). What’s more, ~96.5% of the reflected light is contained within the +1-diffraction order at the design frequency of *f* = 0.8 THz under *y*-polarized excitation, while the other diffraction orders are greatly suppressed with the intensities approaching 0. This slightly improved performance for *y*-polarization is related to the designed supercell that is periodically arranged in the *y*-direction with the period of 90 μm, perfectly mimicking the periodic boundary condition used to calculate the reflection coefficients of the meta-atoms shown in Fig. 1(a).

**Figure 7:**
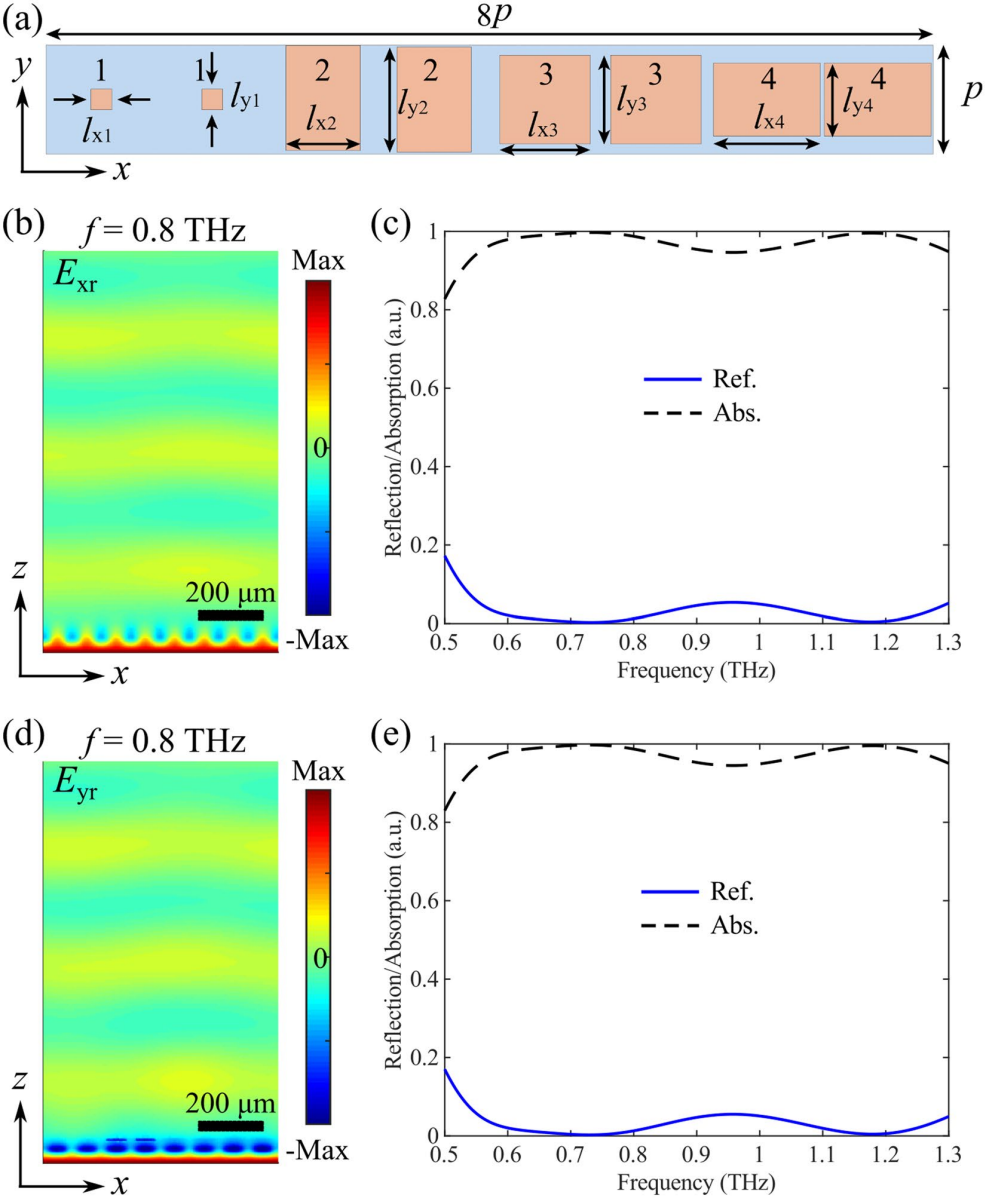
(**a**) Supercell of the polarization-sensitive switchable multifunctional THz metasurface that functions as a polarization-independent broadband absorber. The dimensions are *l*_x1_ = 17 μm, *l*_y1_ = 17 μm, *l*_x2_ = 60.5 μm, *l*_y2_ = 88.4 μm, *l*_x3_ = 73.5 μm, *l*_y3_ = 73.5 μm, *l*_x4_ = 88.4 μm, and *l*_y4_ = 60.5 μm. (**b,c**) Theoretical performance of the metasurface for *x*-polarization, displaying b) the *x*-component of the reflected electric field (*E*_rx_) at the design frequency of *f* = 0.8 THz, and (**c**) reflection and absorption spectra as a function of the frequency. (**d,e**) Theoretical performance of the metasurface for *y*-polarization, displaying d) the *y*-component of the reflected electric field (*E*_ry_) at the design frequency of *f* = 0.8 THz, and (**e**) reflection and absorption spectra as a function of the frequency. VO_2_ is in its insulating state with *σ* = 200 S/m.

Besides polarization-insensitive beam-steering for reflected THz waves, a more desired functionality in practical applications is to achieve polarization-splitting that can anomalously reflected *x*-polarized and *y*-polarized waves into different directions. Therefore, we propose a polarization beam splitter by designing opposite linear phase-gradients for the two incident polarizations since the angle of anomalous reflection is mainly determined by the introduced phase-gradient^5^. Similar to the polarization-insensitive design, here we discretize the reflection phases with a step of π/2, and the contour lines for the *x*- and *y*-polarizations are displayed with solid and dashed black curves in Fig. 4(b). By properly selecting the meta-atoms from the intersection points, four anisotropic VO_2_ bricks are used to construct a supercell with eight meta-atoms, shown in Fig. 7(a). When VO_2_ is in its insulating state at room temperature, all the elements comprising the supercell have sufficiently high absorption, therefore making the metasurface functions as a broadband absorber with high-performance (Fig. 7(b–e)). More importantly, the absorption is independent of the incident polarization since the top VO_2_ antennas have negligible effect in the absorption process. From Fig. 7(c,e), one can clearly see the identical absorption efficiencies, which are consistent with the values in Fig. 5.

**Figure 8:**
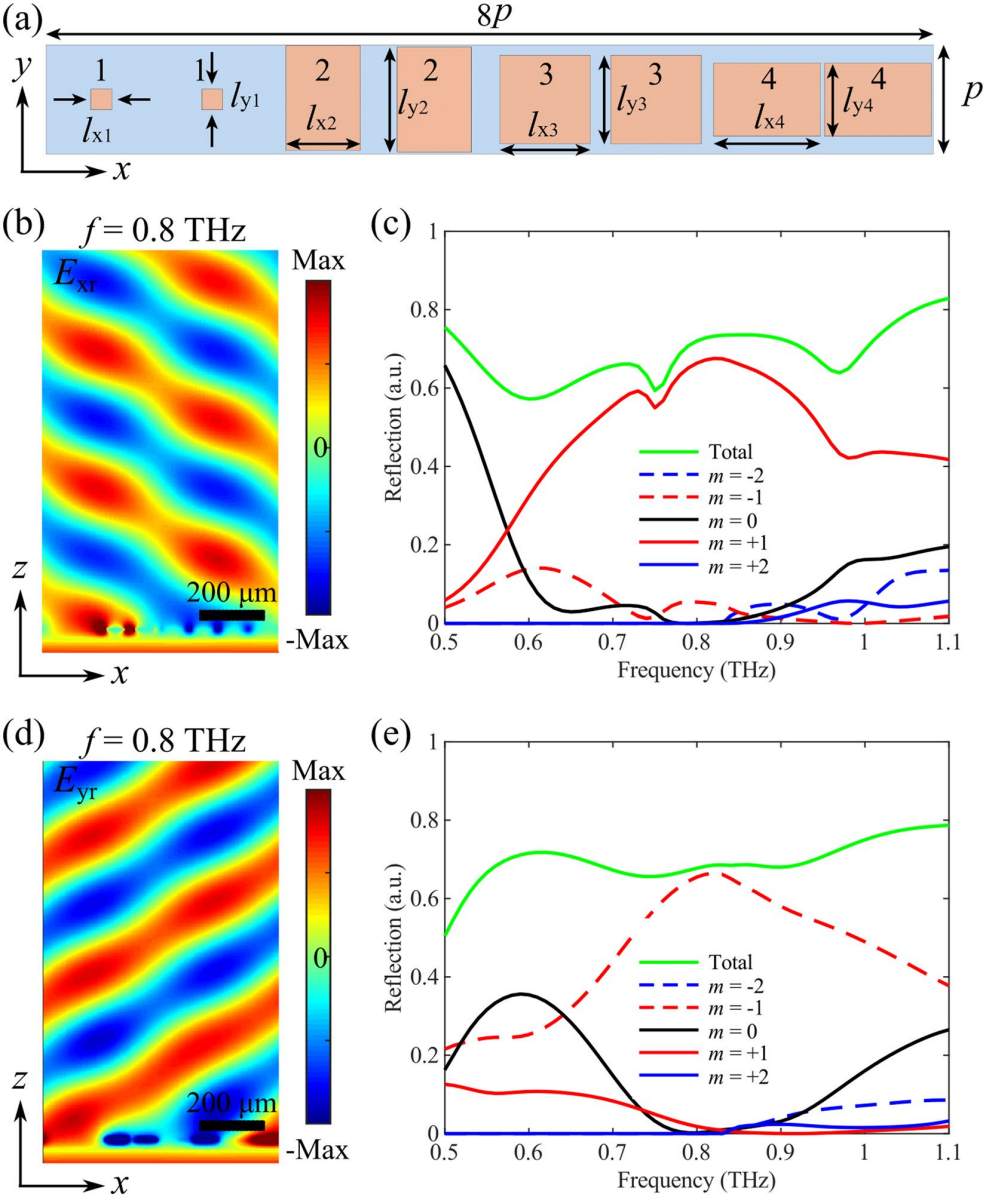
(**a**) Supercell of the polarization-sensitive switchable multifunctional THz metasurface that functions as a broadband polarization-splitter. The dimensions are *l*_x1_ = 17 μm, *l*_y1_ = 17 μm, *l*_x2_ = 60.5 μm, *l*_y2_ = 88.4 μm, *l*_x3_ = 73.5 μm, *l*_y3_ = 73.5 μm, *l*_x4_ = 88.4 μm, and *l*_y4_ = 60.5 μm. The reflection phases of the meta-atoms 1–4 are −15.1° (−15.1°), 74.8° (255.1°), 165.4° (165.4°) and 255.1° (74.8°), respectively, for *x*-polarization (*y*-polarization). (**b,c**) Theoretical performance of the metasurface for *x*-polarization, displaying b) the *x*-component of the reflected electric field (*E*_rx_) at the design frequency of *f* = 0.8 THz, and (**c**) amount of incident THz wave reflected into the different diffraction orders as a function of the frequency. (**d,e**) Theoretical performance of the metasurface for *y*-polarization, displaying (**d**) the *y*-component of the reflected electric field (*E*_ry_) at the design frequency of *f* = 0.8 THz, and (**e**) amount of incident THz wave reflected into the different diffraction orders as a function of the frequency. VO_2_ is in its fully metallic state with *σ* = 2 × 10^5^ S/m.

When VO_2_ is in its fully metallic state, the supercell composed of eight elements supplies a 2π phase span with a constant phase step of ±π/2 for *x*- and *y*-polarizations at *f* = 0.8 THz, resulting in efficient diffractions into ±1 orders with angles of ±31.4°, respectively (Fig. 8). As shown in Fig. 8(b,d), the two orthogonal linear polarizations are reflected at the opposite sides of the surface normal with identical angles, indicating the good performance of polarization-splitting at *f* = 0.8 THz. To further investigate the performance quantitively, we calculate the amount of the reflected THz wave into different orders as a function of the frequency. At the design frequency of *f* = 0.8 THz, ~92.1% and ~96.7% of the reflected THz waves go to the ±1 diffraction orders with the absolute reflectivities reaching 66.4% and 65.6% for *x*- and *y*-polarizations, respectively. When the frequency moves away from the designed value, the capability of polarization-splitting is still good, manifesting the broad operation bandwidth.

## Potential Fabrication Process of the Proposed Structure

**Figure 9:**
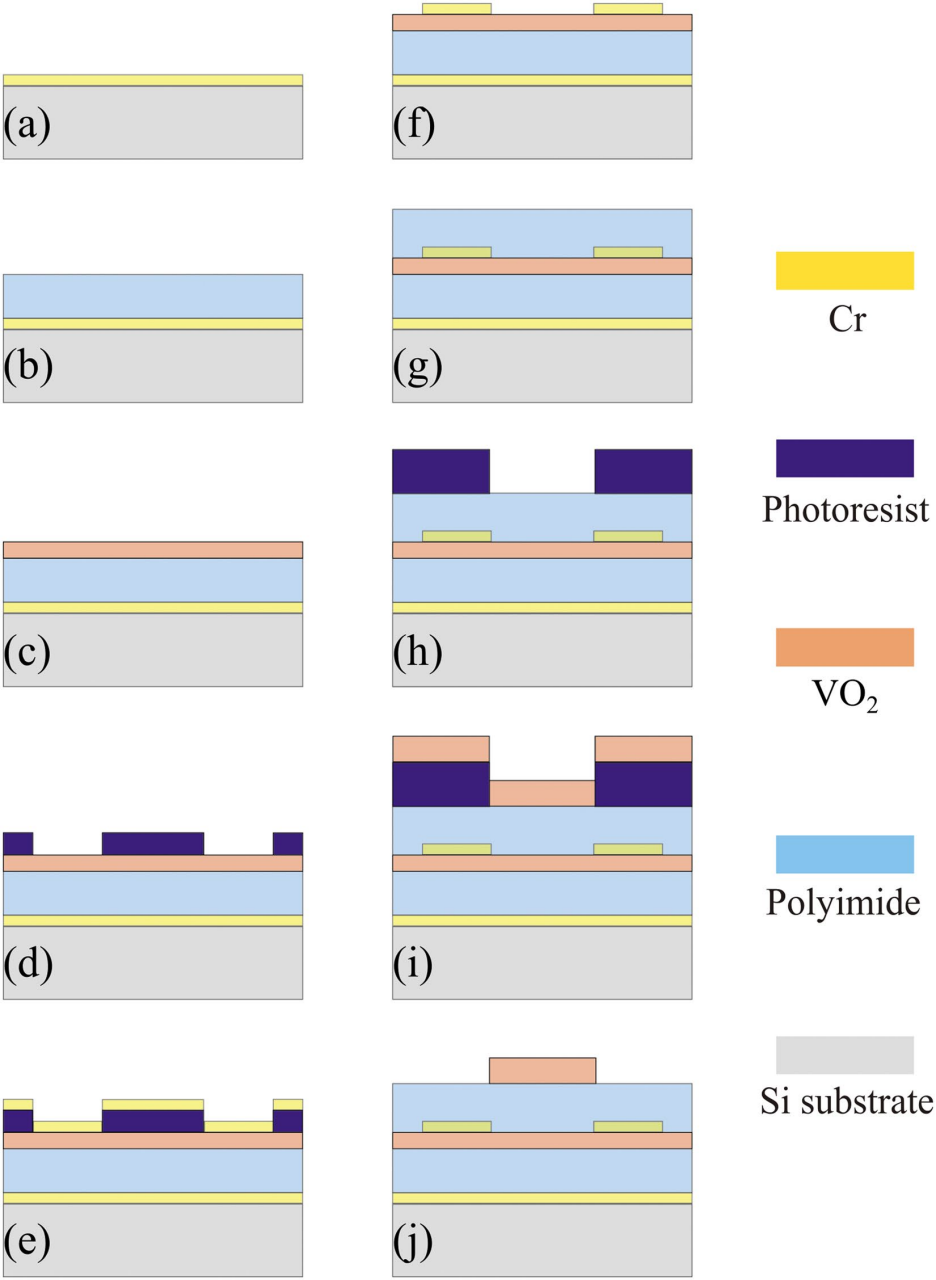
Potential fabrication process of the processed structure: (**a**) 300-nm-thick Cr layer is deposited on double-side-polished Si substrate; (**b**) 30-μm-thick polyimide layer is spin coated and cured at high temperature (~300 °C) (**c**) 1-μm-thick VO_2_ layer is magneton sputtered and annealed at ~450 °C; (d) Photoresist is spin coated and optical lithography; (**e**) Cr is deposed. (**f**) Lift-off process; (**g**) 34-μm-thick polyimide layer is spin coated and cured at high temperature (~300 °C). (**h**) Photoresist is spin coated and optical lithography; (**i**) 1-μm-thick VO_2_ layer is magneton sputtered. (**j**) Lift-off process and the remaining VO_2_ is annealed at ~450 °C. The polyimide can survive at high temperature up to 450 °C.

**Figure 10:**
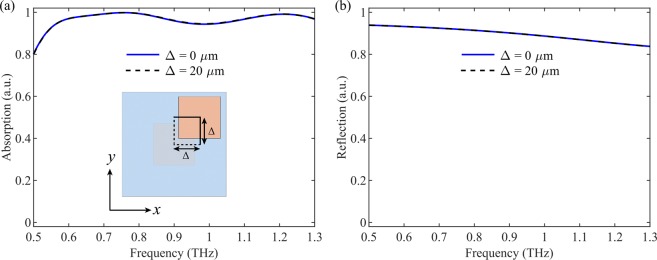
Tolerance analysis when there is multi-layer alignment error. (**a**) Simulated absorption of the homogeneous metasurface at normal incidence when VO_2_ is in its insulating state with *σ* = 200 S/m. (**b**) Simulated reflection of the homogeneous metasurface at normal incidence when VO_2_ is in its fully metallic state with *σ* = 2 × 10^5^ S/m. The inset in (**a**) shows the schematic of the mis-aligned structure. The geometrical parameters are the same as those in Fig. 1.

Regarding the potential fabrication pross of the proposed structure, we could combine the standard ultraviolet lithography with thin-film deposition techniques^29,72^. The fabrication process begins with a double-side-polished silicon (Si) wafer, and the process is presented as Fig. 9: (a) 300-nm-thick Cr layer is deposited on Si substrate using e-beam evaporation; (b) 30-μm-thick polyimide layer is spin coated and cured at ~300 °C; (c) 1-μm-thick VO_2_ layer is magneton sputtered and annealed at ~450 °C; (d) photoresist is spin coated and optical lithography; (e) 300-nm-thick Cr is deposed using e-beam evaporation; (f) the photoresist is dissolved by a lift-off process to form the Cr SRRs; (g) 34-μm-thick polyimide layer is spin coated and cured at ~300 °C; (h) photoresist is spin coated and optical lithography; (i) 1-μm-thick VO_2_ layer is magneton sputtered; (j) the photoresist is dissolved by a lift-off process to form the VO_2_ bricks and the remaining VO_2_ is then annealed at ~450 °C. As a final comment, it should be noted that our device would be insensitive to the proper multi-layer alignment errors during the fabrication since the proposed structure can be regarded as two independent devices isolated by the continuous VO_2_ film when VO_2_ is in different states (Fig. 10).

## Conclusions

In conclusion, we have proposed a type of switchable THz metasurfaces with tunable and diversified functionalities by utilizing the insulator-to-metal transition in VO_2_.

The designed homogeneous metasurface can be dynamically switched from a broadband absorber to an efficient reflector by altering the working temperature. When VO_2_ is in insulating state at room temperature, the metasurface is capable of absorbing the normally incident THz wave in the frequency range of 0.535–1.3 THz with the average absorption of ~97.2%. If the VO_2_ is switched to fully metallic state at high temperature, the designed metasurface exhibits broadband and efficient reflection (>80%) in the frequency range from 0.5 to 1.3 THz. Based on the meta-atom design, we further extend the functionalities by introducing phase-gradients when VO_2_ is in its fully metallic state and consequently achieve polarization-insensitive beam-steering and polarization-splitting, while maintaining broadband absorption in insulating state. It should be emphasized that the achieved two functionalities exhibit comparable and even superior performance once compared with the existing metasurface-based absorbers^72^ and beam-steerers^74–76^, in terms of efficiencies and operating bandwidth. Additionally, the fully insulator-to-metal transition makes VO_2_ an excellent candidate to bridge two isolated devices together, which is hard to achieve with other phase-change materials possessing limited tunability. Although VO_2_ only exhibits volatile switching, it is easier to realize reversible tuning between insulating and metallic states with lower power consumption, superior to GST alloys which need high power density and protective layer. The proposed switchable THz metasurfaces can enable advanced applications in THz society, such as temperature sensor and imaging.

## Data Availability

All relevant data are within the paper.
